# Evaluating the predictive value of initial lactate/albumin ratios in determining prognosis of sepsis patients

**DOI:** 10.1097/MD.0000000000037535

**Published:** 2024-03-22

**Authors:** Jianhua Hu, Qun Jin, Honglong Fang, Weiwen Zhang

**Affiliations:** a Department of Critical Care Medicine, The Quzhou Affiliated Hospital of Wenzhou Medical University, Quzhou People’s Hospital, Quzhou, Zhejiang Province, China.

**Keywords:** lactate/albumin ratio, predictive indicators, prognosis, sepsis

## Abstract

Sepsis remains a significant clinical challenge owing to its complex pathophysiology and variable prognosis. The early identification of patients at a higher risk of poor outcomes can be crucial for improving treatment strategies. This study aimed to evaluate the predictive value of early serum lactate and albumin levels and the lactate/albumin (L/A) ratio for 28-day prognosis in patients with sepsis. Patients diagnosed with sepsis between January 2021 and December 2022 were evaluated using a retrospective cohort methodology. Inclusion followed the International Consensus on sepsis and septic shock (Sepsis-3) guidelines and patients were selected based on well-defined criteria. Variables such as lactate, albumin, and the L/A ratio were documented within the first 24 hours of admission. Statistical analyses were performed using various tools, including the nonparametric Mann–Whitney *U* test and receiver operating characteristic curves. A total of 301 patients were divided into the survival (n = 167) and death (n = 134) groups. Notable differences were detected in the incidence of pulmonary infection, shock, lactate, albumin, and the L/A ratio. The L/A ratio was identified as a key predictor with an area under the curve of 0.868, an optimal cutoff value of >0.17, a sensitivity of 56.21%, and a specificity of 94.18%. Significant disparities in mortality rates and survival times were observed for the lactate, albumin, and L/A levels. This study underscores the predictive value of early serum lactate and albumin levels and the L/A ratio for 28-day prognosis in patients with sepsis, with the L/A ratio showing a superior predictive capability. These findings highlight the importance of L/A ratio as a robust and precise marker for evaluating the future clinical course of patients with sepsis, potentially aiding early detection and management.

## 1. Introduction

Sepsis represents a grave global health challenge and is characterized by life-threatening organ dysfunction stemming from a dysregulated host response to infection, manifesting as an intense systemic inflammatory reaction. Historically known as “septicemia,” it is often solely associated with circulating purulent bacteria and toxins. However, this simplification overlooks the significant systemic response. In reality, less than half of sepsis cases yield positive blood cultures, indicating that bacteremia is not synonymous with sepsis. Systemic manifestations primarily result from various inflammatory mediators. Previous research underscores the role of post-traumatic stress responses in compromising the intestinal mucosal barrier and altering microbiota, culminating in ectopic bacterial colonization and subsequent overwhelming inflammation, leading to organ damage.^[1]^ This highlights the integral role of intestinal factors in the pathogenesis of sepsis. Fundamentally, sepsis is organ dysfunction caused by a deranged host reaction to infection. Alarmingly, sepsis has a global mortality rate of 19.7%,^[2,3]^ emphasizing its complexity. Pursuant to the “Saving Sepsis Campaign: 2021 Guidelines,” there is a pronounced need to enhance diagnostic precision, refine treatment modalities, and boost survival outcomes.^[4]^

Early detection and timely management of biological markers to predict mortality in patients with sepsis are crucial for enhancing prognostic outcomes.^[5]^ Lactate, a product of anaerobic metabolism, often accumulates under hypoxic or shock conditions, indicating metabolic stress in the body. It is notably elevated in sepsis and reflects the severity of the body’s response to infection. Research has demonstrated a significant correlation between elevated lactate levels and sepsis-associated mortality rates, emphasizing its role in the early diagnosis, treatment, and prognosis of this condition. In contrast, serum albumin, a vital plasma protein, maintains colloid osmotic pressure, underpins immune functions, and serves as a carrier for various molecules. Its concentration in the body can reflect a patient’s nutritional, physiological, and inflammatory status. Reduced albumin levels are often associated with illness severity. Recognized as a marker of systemic inflammation, the prognostic capabilities of albumin for sepsis have been well-documented in numerous studies.^[6,7]^

Considering the individual importance of lactate and albumin in sepsis prognosis, their combined assessment through the lactate/albumin (L/A) ratio could potentially offer a composite marker that mirrors both the metabolic and physiological status of septic patients. However, despite the established roles of lactate and albumin, the predictive value of L/A ratio in sepsis prognosis remains relatively uncharted, warranting further investigation. Our study aimed to bridge this knowledge gap by selecting adult sepsis patients to delve deeper into the early predictive capabilities of the L/A ratio, serum lactate, and albumin concerning the 28-day prognosis. By shedding light on these interrelationships, we aspire to enrich the understanding of sepsis dynamics, promoting a more refined approach to its management, and consequently, bolstering survival and recovery rates from this dire condition.

## 2. Materials and methods

### 2.1. Study design

This study implemented a retrospective cohort methodology, focusing on the critical evaluation of patients diagnosed with sepsis at the Quzhou Affiliated Hospital of Wenzhou Medical University. The selection period ranged from January 2021 to December 2022, encapsulating a comprehensive 2-year span to ensure the robustness of the sample. Patients who were subsequently admitted to the intensive care unit for continued treatment were the primary subjects of this study. The integration of an extended timeframe and intensive care unit admission criteria is pivotal in formulating a well-defined participant pool representing the severity and complexity of sepsis treatment in a real-world context. Ethical considerations were meticulously adhered to throughout this study. All participants were informed of the study objectives, procedures, potential risks, and benefits. Following a thorough explanation, informed consent was obtained from each participant to ensure voluntary participation and comprehension of their rights and responsibilities within the study. The research protocol, including the design, methodology, ethical considerations, and analytical procedures, was rigorously reviewed and approved by the Ethics Committee of the Quzhou Affiliated Hospital of Wenzhou Medical University.

### 2.2. Inclusion and exclusion criteria

Patients were included if they were diagnosed with sepsis according to the third edition of the International Consensus on sepsis and septic shock guidelines, were at least 18 years of age, and had complete general information available. The integrity of the sample was further ensured by excluding individuals who were using immunosuppressive agents or steroids, had any hematological diseases, malignant tumors or cachexia, were in the stages of lactation or pregnancy, or had incomplete primary data. This rigorous selection process was integral to the robustness of the study, enabling precise and unbiased evaluation of clinical features, outcomes, and predictive markers in the sepsis patient population.

### 2.3. Data collection

Data collection was an essential phase in this study and was executed with precision using the hospital’s electronic medical record system. Patients with sepsis were systematically identified using the international classification of diseases, eleventh revision codes. Meticulous details, such as sex, age, and comorbidities, were recorded for each patient. Furthermore, critical biochemical parameters, including serum lactate, albumin, C-reactive protein, and procalcitonin levels, were documented within the first 24 hours of admission. These parameters were selected based on their relevance to the pathophysiology of sepsis and potential influence on prognosis. The study was constructed with a clear endpoint marked either by a 28-day period or the unfortunate event of patient death, and prognostic information pertaining to this 28-day interval was systematically documented.

### 2.4. Statistical analysis

Statistical analysis was performed using SPSS software (version 26.0). As continuous variables were non-normally distributed, they were presented as medians (quartiles) [*M (Q*_L_, *Q*_U_)], and intergroup comparisons were conducted using the nonparametric Mann–Whitney *U* test. Categorical variables are expressed as cases (%), and intergroup comparisons were performed using the *χ*^2^ test. Patients were divided into survival and death groups according to their 28-day prognosis. Receiver operating characteristic curves were plotted using GraphPad Prism software (version 9.0). The best cutoff value for predicting sepsis patient mortality was calculated using MedCalc 20.0, along with the area under the curve (AUC), sensitivity, and specificity. The predictive value of the L/A ratio, serum lactate level, and albumin level for 28-day mortality in patients with sepsis was evaluated. Subgroup analysis was performed using the optimal cutoff value. Statistical significance was set at *P* < .05.

## 3. Results

### 3.1. Comparative analysis of general characteristics in sepsis patients across survival and death groups

In our analysis of patients with sepsis, we divided the subjects into 2 distinct groups: those who survived (n = 167) and those who did not survive (n = 134). Our comprehensive examination showed that there were no statistically significant differences between the 2 groups in terms of sex, age, and comorbidities such as diabetes, coronary heart disease, kidney disease, stroke, and liver cirrhosis. However, noticeable differences were detected in the incidence of pulmonary infection and shock, with the death group showing higher percentages (75.4% and 37.3%, respectively) than those in the survival group (53.3% and 15.0%, respectively). Laboratory results also revealed significant disparities in lactate, albumin, L/A, and interleukin-6 levels. Conversely, C-reactive protein and procalcitonin levels were statistically comparable across the groups (Table 1).

**Table 1:** Comparison of general data between sepsis patients in survival and death groups.

Indicators	Survival group (n = 167)	Death group (n = 134)	*χ*^2^/*Z* value	*P* value
Gender [n (%)]			0.012	.912
Male	107 (64.1)	86 (64.2)		
Female	60 (35.9)	48 (35.8)		
Age [yr, *M (Q*_L_, *Q*_U_)]	59 (53, 80)	71 (56, 87)	−2.578	.017
Comorbidities [n (%)]				
Diabetes	37 (22.2)	33 (24.6)	−0.474	.659
Coronary heart disease	26 (15.6)	22 (16.4)	−0.149	.890
Kidney disease	91 (54.5)	73 (54.6)	−0.057	.954
Pulmonary infection	89 (53.3)	101 (75.4)	−4.142	<.001
Stroke	20 (12.0)	22 (16.4)	−1.192	.276
Liver cirrhosis	24 (14.4)	19 (14.2)	−0.140	.896
Shock	25 (15.0)	50 (37.3)	−4.697	<.001
Lab results [*M (Q*_L_, *Q*_U_)]				
Lactate (mmol/L)	2.43 (1.58, 3.51)	5.24 (3.25, 10.15)	−9.196	<.001
Albumin (g/L)	32.58 (27.78, 37.65)	30.45 (23.22, 36.33)	−2.772	.014
L/A	0.09 (0.055, 0.121)	0.20 (0.11, 0.385)	−9.622	<.001
CRP (mg/L)	99.00 (53.71, 99.00)	92.62 (53.27, 99.00)	−0.612	.572
IL-6 (ng/L)	61.47 (27.79, 165.72)	370.70 (107.50, 2540.35)	−8.168	<.001
PCT (μg/L)	3.15 (0.78, 17.36)	5.63 (1.00, 24.42)	−1.979	.070

CRP = C-reactive protein, IL-6 = interleukin-6, L/A = lactate/albumin, PCT = procalcitonin.

### 3.2. Evaluation of lactate, albumin, and L/A ratio as predictive indicators for 28-day prognosis in sepsis

**Figure 1.**
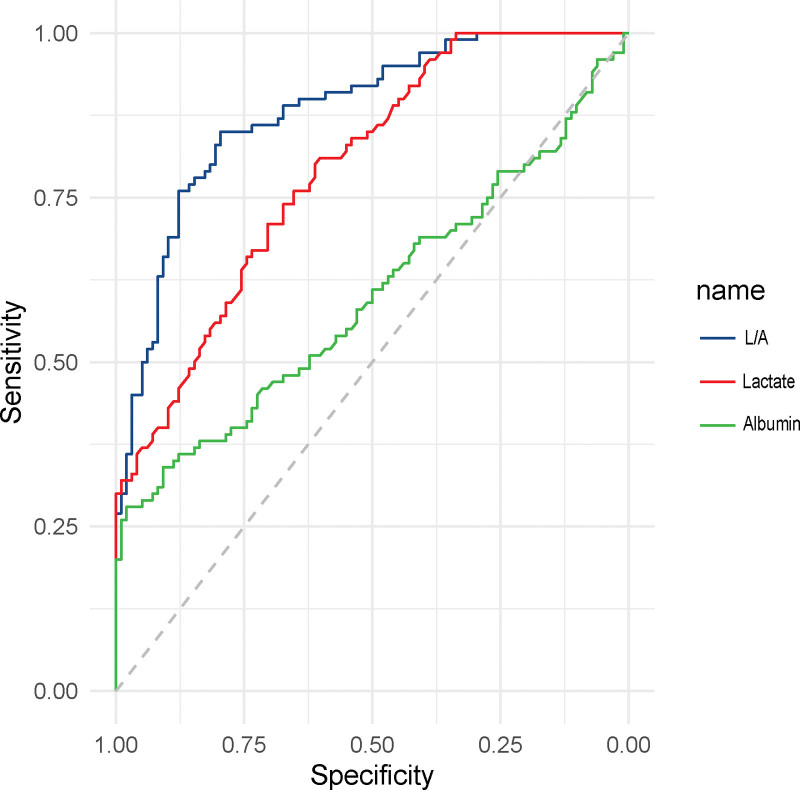
ROC curve for predicting 28-day mortality in sepsis patients based on lactate, albumin, and lactate/albumin levels within 24 hours of admission. ROC = receiver operator characteristic.

The assessment of key variables in forecasting 28-day outcomes in patients with sepsis revealed significant diagnostic insights. In particular, the L/A ratio was an essential predictor, with an AUC of 0.868, 95% CI ranging from 0.764 to 0.861, and a significant *P* value of <0.001. The optimal cutoff value for L/A was identified as >0.17, yielding a Youden index of 0.524, sensitivity of 56.21%, and specificity of 94.18%. Lactate, as an independent variable, demonstrated an AUC of 0.814, with 95% CI between 0.749 and 0.859, and the cutoff value was determined to be >4.22. The corresponding sensitivity and specificity were 59.62% and 91.53%, respectively, indicating that it was a significant indicator with a *P* value of <0.001. Conversely, albumin, although contributing to the predictive model, exhibited a relatively low AUC of 0.598 (95% CI: 0.534–0.655), and the optimal cutoff value was found to be ≤23.11. This resulted in a Youden index of 0.245, sensitivity of 32.26%, and specificity of 91.53%, indicating a *P* value of 0.014 (Table 2, Fig. 1).

**Table 2:** Assessment of lactate, albumin, and lactate/albumin ratio in forecasting 28-day outcome in sepsis patients.

Variable	AUC	95% CI	*P* value	Optimal cutoff	Youden index	Sensitivity (%)	Specificity (%)
Lactate	0.814	0.749–0.859	<.001	>4.22	0.516	59.62	91.53
Albumin	0.598	0.534–0.655	.014	≤23.11	0.245	32.26	91.53
L/A	0.868	0.764–0.861	<.001	>0.17	0.524	56.21	94.18

95% CI = 95% confidence interval, AUC = the area under the receiver operating characteristic curve, L/A = lactate/albumin.

### 3.3. Comparison of 28-day mortality rate and survival time in sepsis patients across different lactate, albumin, and L/A ratio levels

The analysis conducted on a cohort of sepsis patients (Table 3) revealed essential findings related to the 28-day mortality rate and survival time across different L/A ratios, albumin levels, and lactate concentrations. Key observations included a substantial difference in mortality rates and survival times when patients were divided based on the L/A ratio. The group with L/A ≤ 0.17 showed a significantly lower mortality rate of 20.3% (47 cases) compared to the group with L/A > 0.17, with a remarkable rate of 82.9% (58 cases). Survival time in these groups was significantly different, with the lower L/A group having a median of 27.5 days (*Q*_L_ = 20.3, *Q*_U_ = 28.0), and the higher L/A group reporting a survival time median of 0.9 days (*Q*_L_ = 0.9, *Q*_U_ = 2.9). Both mortality rate and survival time in these groups were statistically significant (*P* < .001). Similarly, albumin levels also demonstrated significant variations, with the ≤23.11 g/L group showing a higher mortality rate (63.5%, 33 cases) and a shortened median survival time of 3.6 days (*Q*_L_ = 0.9, *Q*_U_ = 24.3) compared to the albumin >23.11 g/L group. Both factors were statistically significant (*P* < .001). The division based on the lactate concentration also revealed significant differences. The >4.22 mmol/L group reported a higher mortality rate of 69.0% (60 cases) and a drastically reduced median survival time of 0.9 days (*Q*_L_ = 0.9, *Q*_U_ = 3.6) compared to the lactate ≤4.22 mmol/L group. Again, these findings were statistically significant (*P* < .001).

**Table 3:** Comparison of 28-day mortality rate and survival time in sepsis patients across different lactate, albumin, and lactate/albumin ratio levels.

Group	Number of cases	28-day mortality rate [% (cases)]	*χ*²/*Z* value (mortality)	*P* value (mortality)	Survival time [d, *M* (Q_L_, Q_U_)]	*χ*²/*Z* value (survival)	*P* value (survival)
L/A ≤ 0.17 group	231	20.3 (47)	78.890	<.001	27.5 (20.3, 28.0)	116.029	<.001
L/A > 0.17 group	70	82.9 (58)			0.9 (0.9, 2.9)		
Albumin ≤23.11 g/L group	52	63.5 (33)	24.084	<.001	3.6 (0.9, 24.3)	47.097	<.001
Albumin >23.11 g/L group	249	27.7 (69)			27.5 (2.7, 28.0)		
Lactate >4.22 mmol/L group	87	69.0 (60)	73.340	<.001	0.9 (0.9, 3.6)	110.602	<.001
Lactate ≤4.22 mmol/L	214	20.5 (44)			27.5 (21.2, 28.0)		

L/A = lactate/albumin.

## 4. Discussion

Clinical management of sepsis remains an intricate challenge, necessitating nuanced strategies for diagnosis, prognosis, and therapeutic intervention. Our research, based on a meticulous analysis of sepsis patients, has unveiled a series of pivotal insights that can potentially reshape the current paradigms in sepsis management. The L/A ratio is a robust predictive marker for 28-day mortality, outperforming the prognostic capacity of either lactate or albumin when assessed independently. The superior specificity and sensitivity indices associated with the L/A ratio underscore its potential as an indispensable tool for the early prognosis of sepsis. By discerning this ratio, clinicians can refine their therapeutic interventions, enabling a more personalized patient management trajectory. Moreover, our study reaffirms the multifaceted nature of sepsis, where variables such as pulmonary infection and shock prevalence, although clinically evident, were distinctly amplified in the death cohort. The results of this study highlight the clinical importance of continuous exploration and integration of novel markers, such as the L/A ratio, fortifying the precision medicine armamentarium against sepsis. In summary, the elucidation of the prognostic value of the L/A ratio offers clinicians a potent tool for enhancing diagnostic accuracy, streamlining therapeutic strategies, and ultimately augmenting survival probabilities in sepsis.

Serum albumin has a negative charge, is weakly acidic, and has a half-life of approximately 18 days. It constitutes the highest percentage of total protein in the plasma (40%–60%) and plays several physiological roles, including maintaining osmotic pressure stability, transport function, providing energy during severe consumption, maintaining acid-base balance, vascular endothelial function barrier, antioxidation, and participation in coagulation functions.^[8]^ Albumin synthesis can be influenced by various factors such as inflammation, malnutrition, and cirrhosis. As an acute-phase negative protein, serum albumin can serve as a prognostic biomarker in patients with sepsis.^[9]^ Some studies have demonstrated that albumin levels <28 g/L are an independent risk factor for sepsis mortality, and low albumin levels increase the sepsis mortality rate.^[10]^ Our study also revealed that sepsis patients with albumin ≤23.11 g/L within 24 hours of admission had a significantly increased 28-day mortality rate, supporting previous conclusions. Thus, albumin ≤23.11 g/L can be considered as an early indicator of sepsis prognosis.

Sepsis is a severe and complex medical condition that can lead to multi-organ dysfunction, representing a critical state of immune dysfunction and catabolic metabolism. This dysfunction ultimately leads to mortality. Early evaluation and intervention have been shown to decrease the likelihood of its progression into severe infection.^[11,12]^ In this study, we further investigated the early prognostic value of L/A, lactate, and albumin levels in adult patients with sepsis. The objective was to enhance early assessment and intervention and consequently reduce mortality rates. Our study clearly demonstrated that the differences in lactate, albumin, and the L/A ratio within 24 hours of admission between the survival and death groups of patients with sepsis were statistically significant. Receiver operator characteristic curve analysis for 28-day mortality prediction revealed that the AUC for lactate, albumin, and L/A were 0.814, 0.598, and 0.868, respectively. The sensitivity was 59.62% for lactate, 32.26% for albumin, and 56.21% for L/A, while the specificity was 91.53% for lactate and albumin, and 94.18% for L/A. Additionally, a higher 28-day mortality rate was observed in sepsis patients with L/A > 0.17 compared than in those with L/A ≤ 0.17. These findings underscore that the early L/A ratio has superior prognostic evaluation potential compared with lactate and albumin levels individually. The predictive value of the L/A ratio in the early stages of sepsis makes it a valuable indicator that could serve as a vital tool for early prognosis of sepsis. Therefore, the integration of the L/A ratio into clinical practice may present a refined approach towards patient management, contributing to more precise therapeutic decisions and potentially improving patient outcomes.

Lactate is generated in the body owing to hypoxia or insufficient tissue perfusion. During anaerobic glycolysis, pyruvate dehydrogenase fails to convert pyruvate into acetyl coenzyme A in a timely manner, leading to significant production of pyruvate. Initially, this consumes 2 molecules of adenosine triphosphate and eventually generates 4 adenosine triphosphate molecules, which are subsequently reduced to lactate-by-lactate dehydrogenase, where it starts to accumulate.^[13]^ Elevated lactate levels have been correlated with mortality and are widely used for early diagnosis, management, and risk stratification in patients with septic shock.^[14,15]^ However, elevated lactate levels can also be influenced by various factors, including medication, liver or kidney dysfunction, diabetic ketoacidosis, malignancy, etc.^[16,17]^ The latest sepsis guidelines consider lactate levels ≥4 mmol/L as a reference for poor prognosis in patients with septic shock. Our findings indicated that sepsis patients with lactate levels >4.22 mmol/L within 24 hours of admission had a significantly increased 28-day mortality rate, consistent with the guidelines’ conclusions. Therefore, early lactate levels of >4.22 mmol/L can be considered an indicator of prognosis in sepsis and should be closely monitored by clinicians.

Our aim was to elucidate the prognostic potential of the L/A ratio in sepsis. Both our study and that of Li et al^[18]^ emphasized the prognostic significance of the L/A ratio in sepsis. Although Li et al incorporated additional variables, notably interleukin-6, and achieved a slightly higher AUC, our study offered a focused analysis solely on early serum markers, asserting the paramount importance of L/A in predicting 28-day sepsis outcomes. Similarly, Kabra et al^[19]^ reported high specificity and sensitivity for L/A, which is consistent with our findings. However, our study provides a comprehensive evaluation, leveraging a larger sample size and a broader time frame, ensuring more robust and generalizable conclusions regarding the predictive capacity of the L/A ratio in sepsis prognosis. Based on previous studies, Chen et al^[20]^ emphasized the clinical significance of the L/A ratio in predicting sepsis outcomes. However, while Chen et al introduced the Lac/Alb × age score as an innovative prognostic metric, our study focused on the standalone values of early serum Lac/Alb, lactate, and albumin levels. Notably, our research provides a more contemporary cohort, rigorous statistical analyses, and an in-depth evaluation of the first 24-hour metrics, augmenting the significance of the Lac/Alb ratio in early sepsis management and prognosis. These shared insights and variances across studies accentuate the evolving understanding of sepsis biomarkers and underscore the importance of continued research in this domain.

In addition to the markers we emphasized, other prognostic indicators could augment the understanding of sepsis management, and warrant investigation in future studies. The single-center nature of our data source is a notable limitation, introducing potential biases inherent to the patient demographics and management protocols of that particular center. Implementing multicenter studies would undeniably introduce new challenges such as variations in clinical practices, patient populations, and diagnostic criteria across centers, requiring meticulous standardization. To advance our understanding of the significance of the L/A ratio in sepsis, future studies should encompass prospective studies or randomized controlled trials. Such studies might offer a more definitive insight into the utility of the L/A ratio in sepsis management while simultaneously addressing the limitations and biases associated with retrospective, single-center designs.

## 5. Conclusions

The study revealed that early serum lactate, albumin, and the L/A have predictive values for 28-day prognosis in patients with sepsis. Among these indicators, the L/A ratio demonstrated superior predictive capability compared with lactate and albumin alone. This finding suggests a potentially vital tool for clinicians in early detection and management, emphasizing the importance of the L/A ratio as a robust and precise marker for evaluating the future clinical course of patients with sepsis.

## Acknowledgments

We express our gratitude for the technical assistance provided by our colleagues, Danqiong Wang and Jian Luo, who provided valuable suggestions for academic research. The participation and informed consent extended by the patients for this investigation are highly appreciated.

## Author contributions

**Conceptualization:** Jianhua Hu.

**Data curation:** Jianhua Hu.

**Investigation:** Qun Jin, Honglong Fang.

**Methodology:** Qun Jin, Honglong Fang.

**Visualization:** Weiwen Zhang.

**Writing – original draft:** Jianhua Hu.

**Writing – review & editing:** Weiwen Zhang.
